# Demographic bias of expert-level vision-language foundation models in medical imaging

**DOI:** 10.1126/sciadv.adq0305

**Published:** 2025-03-26

**Authors:** Yuzhe Yang, Yujia Liu, Xin Liu, Avanti Gulhane, Domenico Mastrodicasa, Wei Wu, Edward J. Wang, Dushyant Sahani, Shwetak Patel

**Affiliations:** ^1^ Department of Electrical Engineering and Computer Science, Massachusetts Institute of Technology, Cambridge, MA, USA.; ^2^ Department of Electrical and Computer Engineering, University of California, San Diego, La Jolla, CA, USA.; ^3^ Paul G. Allen School of Computer Science & Engineering, University of Washington, Seattle, WA, USA.; ^4^ Department of Radiology, University of Washington School of Medicine, Seattle, WA, USA.; ^5^ OncoRad/Tumor Imaging Metrics Core (TIMC), Department of Radiology, University of Washington, Seattle, WA, USA.; ^6^ Department of Electrical and Computer Engineering, University of Washington, Seattle, WA, USA.

## Abstract

Advances in artificial intelligence (AI) have achieved expert-level performance in medical imaging applications. Notably, self-supervised vision-language foundation models can detect a broad spectrum of pathologies without relying on explicit training annotations. However, it is crucial to ensure that these AI models do not mirror or amplify human biases, disadvantaging historically marginalized groups such as females or Black patients. In this study, we investigate the algorithmic fairness of state-of-the-art vision-language foundation models in chest x-ray diagnosis across five globally sourced datasets. Our findings reveal that compared to board-certified radiologists, these foundation models consistently underdiagnose marginalized groups, with even higher rates seen in intersectional subgroups such as Black female patients. Such biases present over a wide range of pathologies and demographic attributes. Further analysis of the model embedding uncovers its substantial encoding of demographic information. Deploying medical AI systems with biases can intensify preexisting care disparities, posing potential challenges to equitable healthcare access and raising ethical questions about their clinical applications.

## Teaser

Compared to certified radiologists, expert-level AI models show notable and consistent demographic biases across pathologies.

## INTRODUCTION

Artificial intelligence (AI) has increasingly been deployed in real-world clinical settings, especially for medical imaging (*1*–*4*). The latest developments include vision-language foundation models that operate on a self-supervised learning paradigm (*5*, *6*), eliminating the need for explicit pathology annotations while maintaining human-level diagnostic accuracy across various modalities and disease conditions (*5*, *7*–*9*). Notably, in radiology, by simultaneously using image and text inputs and leveraging the information naturally present in clinical reports associated with radiology images, foundation models identify pathologies without dependence on specific annotations, achieving performance that matches the expertise of radiologists and, in some cases, surpasses the expected diagnostic benchmarks (*10*, *11*).

Despite the plausible performance in diagnosing unseen pathologies (*10*), the foundation model could amplify existing biases in the data, causing diagnosis disparities across protected subpopulations and leading to unequal predictive outcomes for specific demographics (*12*–*14*) (e.g., discrepancies in diagnosis rates between Black and white patients). Existing literature has revealed that chest x-ray classifiers trained to predict the presence of disease systematically underdiagnosed Black patients (*12*, *14*), potentially leading to incorrect triage decisions and delayed medical treatment. Although algorithmic biases have been studied in the supervised setting (*14*–*16*) (e.g., models trained for specific diseases like “no finding”) or image-only foundation model (*17*) (e.g., pretrained solely on chest x-rays and fine-tuned on labeled data), little attention has been paid to vision-language foundation models. These models, notably, free from explicit supervision through multimodal training and zero-shot inference, theoretically have reduced potential to inherit human labeling biases. However, to ensure the responsible and fair deployment, it is essential to investigate potential biases these models may have, understand the sources and measure their outcomes, and where possible, initiate corrective actions (*18*).

**Fig. 1.**
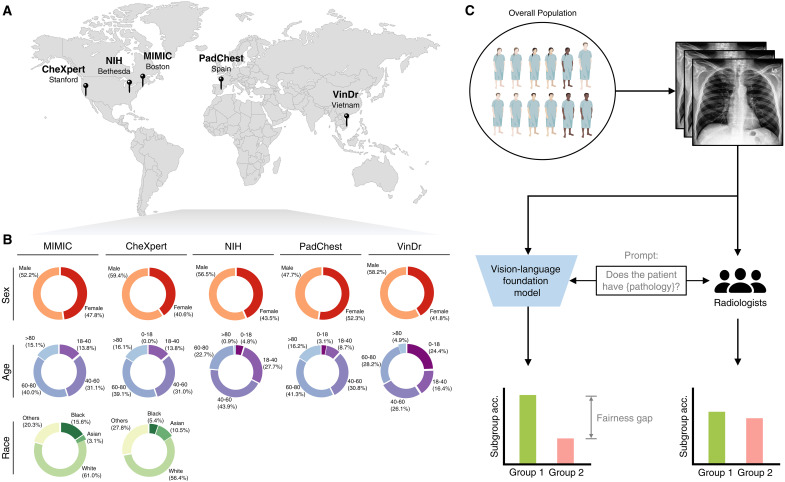
The model evaluation pipeline. (**A**) We use internationally sourced chest x-rays datasets for model evaluation, including MIMIC (Boston, MA), CheXpert (Stanford, CA), NIH (Bethesda, MD), PadChest (Spain), and VinDr (Vietnam). (**B**) Distribution of demographics attributes (i.e., sex, age, and race) of each dataset. For each attribute, we select common subgroups based on literature definition (sex: male and female; age: 0 to 18, 18 to 40, 40 to 60, 60 to 80, and >80; race: Asian, Black, white, and others). Each dataset encompasses different proportions of subgroups, reflecting the diverse distributions in real-world clinical settings. (**C**) For fairness evaluation, we processed radiographs through foundation models, accompanied by specific text prompts (e.g., “Does the patient have {pathology}?”; details in Materials and Methods). The evaluations are conducted across a wide range of different pathologies. Concurrently, board-certified radiologists independently reviewed identical subsets of the data, providing diagnoses that served as human fairness evaluations and comparisons (Fig. 2). In addition, we performed evaluations to assess the prediction of demographic attributes (e.g., sex, age, and race) by both the model and three board-certified radiologists, following the same pipeline with modified prompts (Fig. 5). Created in BioRender. Yang (2025) https://BioRender.com/l87p976.

We present a systematic study to measure and understand biases in vision-language foundation models. Using chest x-rays as a driving example, we mainly use CheXzero (*10*), a state-of-the-art self-supervised foundation model in medical imaging, to assess bias and fairness across a broad spectrum of pathologies with demographic subpopulations present in the testing data. We also test another vision-language foundation model (*11*) and show similar findings (fig. S1). Our analysis incorporates five diverse, globally sourced radiology datasets: Medical Information Mart for Intensive Care (MIMIC) (*19*), CheXpert (*20*), National Institutes of Health (NIH) (*21*), PadChest (*22*), and VinDr (*23*). We evaluate fairness within both individual and intersectional subpopulations spanning demographic attributes including race, sex, and age (*12*, *14*). We further compare fairness outcomes of the model with board-certified radiologists, uncovering that the foundation model demonstrates more substantial fairness discrepancies compared to human experts (Fig. 1). Further investigation in direct assessment of demographic attributes from chest x-rays shows that the model exhibits enhanced capacity to predict sensitive demographic information (e.g., age and race) compared to radiologists. Our international evaluation highlights pronounced biases within foundation models contrasted with evaluations by board-certified radiologists, shedding light on the origins of these biases and potential methodologies for bias auditing and correction before real deployments.

## RESULTS

### Datasets, model, and evaluation protocols

We collect five public chest x-ray datasets from diverse global sources. These datasets, as detailed in Table 1, encompass MIMIC (*19*) (357,167 images from 61,927 patients), CheXpert (*20*) (223,458 images from 64,925 patients), and NIH (*21*) (112,120 images from 30,805 patients) from the United States, PadChest (*22*) (160,736 images from 67,590 patients) from Spain, and VinDr (*23*) (5323 images from 5323 patients) from Vietnam. The datasets provide chest x-ray images along with pathology labels and demographic data derived from the respective patients. Both MIMIC and CheXpert present demographic information including sex, age, and race. The remaining datasets (i.e., NIH, PadChest, and VinDr) present demographic details regarding sex and age, with no information available on the race of the patients.

**Table 1.** Characteristics of the datasets used in this study.

		MIMIC	CheXpert	NIH	PadChest	VinDr
	Location	Boston, MA	Stanford, CA	Bethesda, MD	Alicante, Spain	Hanoi, Vietnam
	# Images	357,167	223,458	112,120	160,736	5323
	# Patients	61,927	64,925	30,805	67,590	5323
	# Frontal	230,406	191,014	112,120	111,187	5323
	# Lateral	126,761	32,444	0	49,549	0
	# Pathologies	14	14	15	174	27
Sex	Female	170,698	90,833	48,780	79,880	2227
	Male	186,469	132,625	63,340	80,856	3096
Race	Asian	11,121	23,384	-	-	-
	Black	55,611	11,999	-	-	-
	White	218,037	125,990	-	-	-
	Other	72,398	62,085	-	-	-
Age	0–18	0	103	5402	5529	1298
	18–40	49,353	30,808	31,037	14,033	874
	40–60	111,055	69,245	49,243	41,406	1390
	60–80	142,824	87,378	25,419	62,206	1501
	80–100	53,935	35,924	1019	37,562	260

We use a state-of-the-art self-supervised foundation model in medical imaging, CheXzero (*10*), as a driving example to study fairness of foundation models. The details about model architecture, prompt design, and evaluation protocols are in Materials and Methods. Note that the model was trained in a self-supervised way without using any pathology labels or annotations. We also tested on another vision-language foundation model (*11*) and observed similar findings (fig. S1). We evaluated the model on internationally sourced chest x-rays datasets. In particular, CheXpert, PadChest, and VinDr contain gold-standard ground-truth radiologist labels. Among these datasets, CheXpert test set (666 samples) and VinDr (5323 samples) provide external annotations from three board-certified radiologists, which were used to benchmark the performance and fairness of radiologists’ assessments compared to the model.

To assess the model prediction fairness, we focus on three demographic attributes: sex, age, and race, and dissect the performance of the model within different subpopulations, such as female or Black patients, and the intersectional groups like Black female patients. We follow the literature to examine the class-conditioned error rate that is likely to lead to worse patient outcomes for a screening model (*12*, *14*). For all potential pathology labels, a false negative indicates falsely predicting someone to be healthy when they are ill, which could lead to delays in treatment (*14*) (i.e., an underdiagnosis). Therefore, we evaluate the differences in false-negative rate (FNR) between demographic subpopulations. For “no finding,” we evaluate the false-positive rate (FPR) for the same reason. Equality in these metrics can be viewed as instances of equal opportunity between subgroups (*24*). We then denote the differences in FNR/FPR for two selected subgroups (e.g., Black and white patients) as the underdiagnosis disparity.

### Substantial fairness disparities in foundation model compared to radiologists

**Fig. 2.**
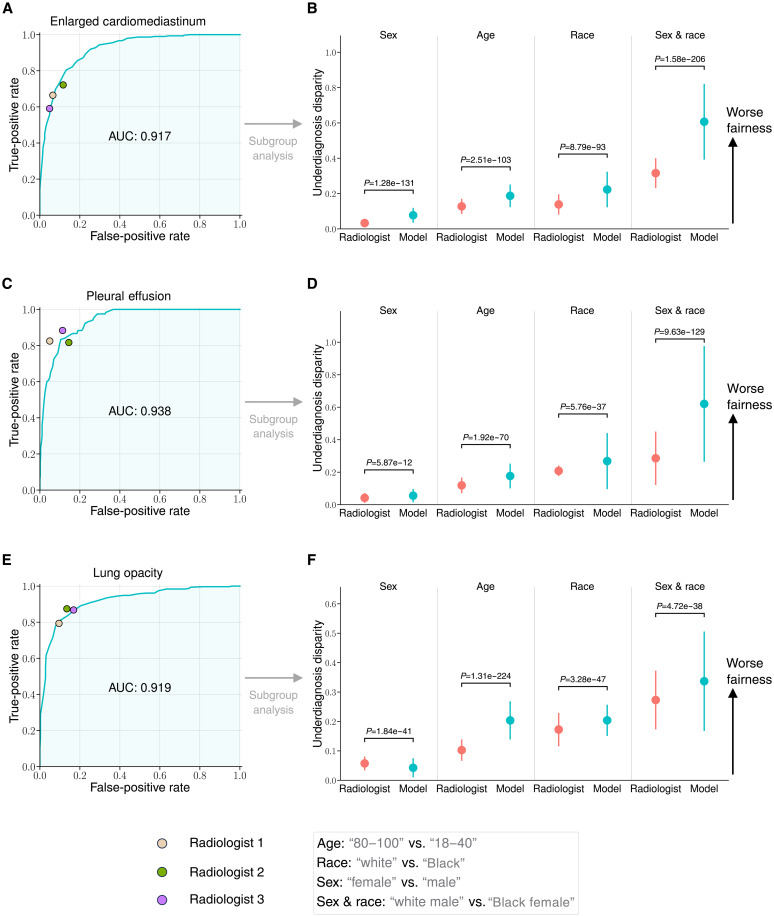
Comparisons of diagnosis AUROC and underdiagnosis disparity for the vision-language foundation model and board-certified radiologists. (**A**, **C**, and **E**) Comparison of the ROC curve of the vision-language foundation model to benchmark radiologists against the test-set ground truth on the CheXpert dataset (*n* = 666). The model outperforms the radiologists when the ROC curve lies above the radiologists’ operating points. The model has an AUC of 0.917 [95% CI (0.905 to 0.928)] for enlarged cardiomediastinum, an AUC of 0.938 [95% CI (0.922 to 0.950)] for pleural effusion, and an AUC of 0.919 [95% CI (0.904 to 0.933)] for lung opacity. (**B**, **D**, and **F**) Comparison of the underdiagnosis disparity of the vision-language foundation model against three board-certified radiologists on the CheXpert test set (*n* = 666). We average the assessments from different radiologists as the evaluation of human biases. The model exhibits significantly higher underdiagnosis bias than that of radiologists on all three pathologies. Error bars indicate 95% CIs estimated using nonparametric bootstrap sampling (*n* = 1000). More results can be found in the fig. S2.

We assess the model’s underdiagnosis disparity across datasets and demographic populations. Since external radiologist annotations are available in certain datasets (i.e., CheXpert and VinDr), we directly compared the overall performance and the performance for subpopulations between the model and radiologists. Figure 2 presents the diagnostic performance and fairness of the vision-language foundation model in contrast to that of board-certified radiologists on the CheXpert dataset (*n* = 666). First, Fig. 2 (A, C, and E) shows the comparison of the receiver operating characteristic (ROC) curves of the model to the operating points of radiologists for three different pathologies. Notably, the model exhibits comparable or better diagnostic performance compared to radiologists [“enlarged cardiomediastinum:” the area under the ROC curve (AUC) = 0.917 and 95% confidence interval (CI) (0.905 to 0.928); “pleural effusion:” AUC = 0.938 and 95% CI (0.922 to 0.950); and “lung opacity:” AUC = 0.919 and 95% CI (0.904 to 0.933)].

In the meantime, we further assess the underdiagnosis disparity between subgroups, which measures the disparity of FNR between two selected subgroups in each category (“female” versus “male” in sex, “80 to 100” versus “18 to 40” in age, “white” versus “Black” in race, and “white male” versus “Black female” in the intersectional group of sex and race). We average the assessments from different radiologists as the evaluation of human biases. When computing FNR for the model, we use the optimal threshold computed on the validation set that maximizes the Youden’s *J* statistic (*25*). Figure 2 (B, D, and F) shows that the model exhibits much larger fairness gaps compared to radiologists, especially for intersectional subgroups. For instance, the model exhibits significantly higher underdiagnosis rate for “enlarged cardiomediastinum” in sex (*P* = 1.28 × 10^−131^, one-tailed Wilcoxon rank-sum test; same test for following attributes), age (*P* = 2.51 × 10^−103^), race (*P* = 8.79 × 10^−93^), and the intersectional of sex and race (*P* = 1.58 × 10^−206^). More results can be found in the fig. S2, including the analysis of other pathologies in CheXpert, and on another dataset from a different site (VinDr). Overall, the model exhibits expert-level pathology detection accuracy, but shows consistently higher underdiagnosis bias compared to radiologists.

### Diagnosis bias in marginalized populations and intersectional groups

**Fig. 3.**
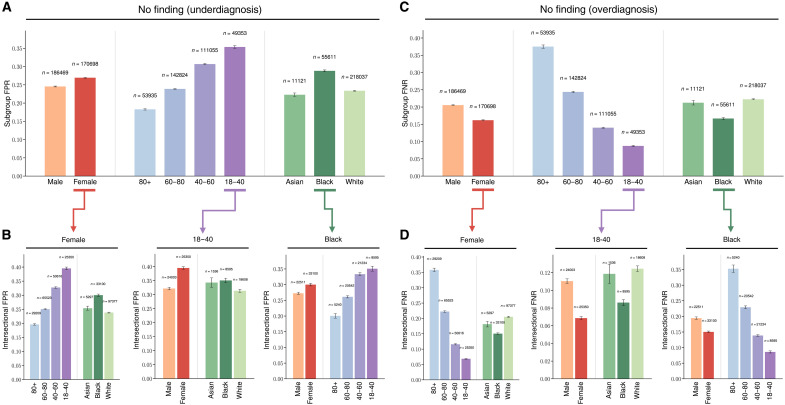
Analysis of underdiagnosis and overdiagnosis across subgroups of sex, age, race, and intersectional groups in the MIMIC dataset. (**A**) The underdiagnosis rate, as measured by the no finding FPR, in the indicated patient subpopulations. (**B**) Intersectional underdiagnosis rates for female patients, patients aged 18 to 40 years, and Black patients. (**C** and **D**) The overdiagnosis rate, as measured by the no finding FNR in the same patient subpopulations as in [(A) and (B)]. Error bars indicate 95% CIs estimated using nonparametric bootstrap sampling (*n* = 1000). More results can be found in the figs. S3 and S4.

We further evaluate the diagnosis bias of the model on MIMIC, the largest and the most diverse chest x-ray dataset in our study. We focus on the no finding label and show both underdiagnosis and overdiagnosis bias of the model on individual and intersectional subpopulations (Fig. 3). FPR is used for assessing underdiagnosis, whereas FNR is used for overdiagnosis. Figure 3A shows substantial fairness gaps between patient subpopulations in each category, especially between the age subgroups “>80” (*n* = 53,935) and “18 to 40” (*n* = 49,353). Moreover, larger gaps of the underdiagnosis rate between the intersectional subgroups can be observed in Fig. 3B. For instance, around 20% FPR discrepancies exist between female patients aged above 80 (*n* = 29,209) and those in their 18 to 40 (*n* = 25,350). Similar observations hold for overdiagnosis (Fig. 3, C and D), the gaps become more notable between intersectional subgroups. The FPR (Fig. 3A) and FNR (Fig. 3C) for no finding shows an inverse relationship across different marginalized subgroups in the CXR dataset. Such an inverse relationship also exists for intersectional subgroups (Fig. 3, B and D) and is consistent across other datasets.

We observe that female patients, patients aged between 18 and 40 years, and Black patients have higher rates of algorithmic underdiagnosis, indicating that these subgroups are most likely being falsely diagnosed as healthy by the model and failing to receive appropriate clinical treatments. Further investigations on intersectional subpopulations reveal that the underdiagnosis rates increased substantially for specific groups of patients, such as Black Female patients. We show in fig. S3 that the observations hold across different pathologies such as “lung opacity” or “pneumonia.” We further confirm in fig. S4 that the disparities remain consistent when tested on external datasets such as CheXpert, NIH, and VinDr.

### Demographic bias in unseen radiographic findings

**Fig. 4.**
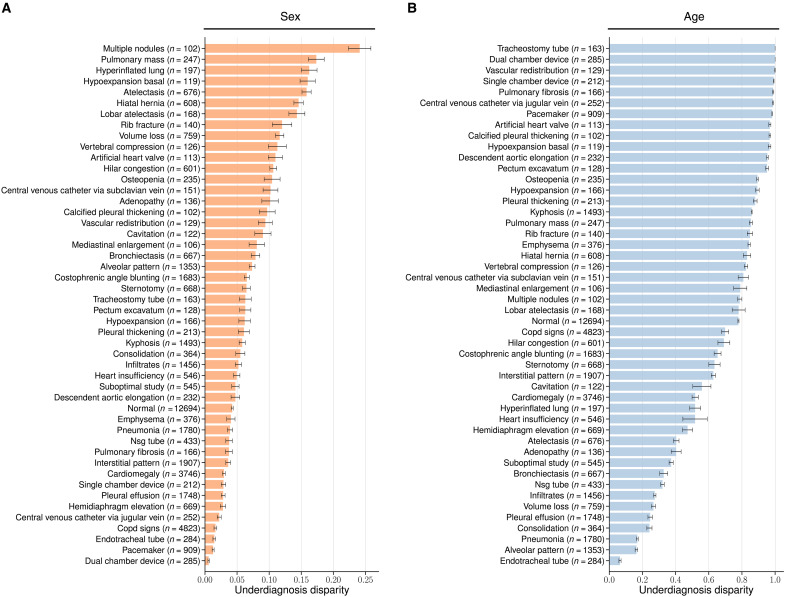
Demographic fairness on unseen radiographic findings in the PadChest dataset. Average underdiagnosis disparity and 95% CI are shown for each radiographic finding (*n* > 100) labeled as high importance by an expert radiologist. (**A**) Underdiagnosis disparity for sex (between group female and male). (**B**) Underdiagnosis disparity for age (between group 18 to 40 and >80. We externally validated the model’s fairness when testing on different data distributions by evaluating model performance on the human-annotated subset of the PadChest dataset (*n* = 39,053). No labeled samples were seen during training for any of the radiographic findings in this dataset.

We extended our analysis to investigate the demographic biases using a much larger and diverse set of pathology labels. We tested the foundation model on the PadChest dataset collected from a different country with 174 radiographic findings and 19 differential diagnoses (*22*). We filtered out 48 radiographic findings where *n* > 100 and the model achieved an AUC of at least 0.7 in the PadChest test set (*n* = 39,053) to further assess the demographic fairness of the model on unseen radiographic findings (*10*). Figure 4 reveals distinct disparities in both sex (female versus male subgroup) and age (>80 versus 18 to 40 subgroup) among those radiographic findings. The maximum underdiagnosis disparity (i.e., “multiple nodules,” *n* = 102) between female and male patients is 24.1% [95% CI (22.5 to 26.0%)], whereas 31 of 48 findings exhibit a fairness gap larger than 5% (Fig. 4A). The discrepancies become even more significant for age, with a 100% fairness gap for “tracheostomy tube” (*n* = 163) between 18 to 40 and >80 subgroups, and 45 of 48 findings exhibit a fairness gap larger than 20% (Fig. 4B).

### Demographic information encoding in foundation model beyond human levels

With consistent demographic bias across international evaluation, we aim to further dissect and explain the performance of the model. Inspired by recent works on algorithmic encoding of demographic information by deep learning models (*26*–*28*), we investigated whether the model encodes demographic information by examining the predictability of sensitive attributes by both the self-supervised foundation model and board-certified radiologists. We selected 480 chest x-ray samples from the MIMIC dataset, ensuring an equal number of samples across all subgroups in three key attributes: sex, age, and race (details in Materials and Methods). Instead of focusing on pathology prediction, we assessed how much the model encodes demographic information by training a linear attribute prediction head using logistic regression on top of the penultimate layer of the model, with the model weights frozen. In the meantime, we involved three board-certified radiologists with over 10 years of experience in chest imaging to label the demographic attributes (sex, age, and race) for the same set of patients based solely on their chest x-rays (details in Materials and Methods). Each radiologist was blinded to the demographic attributes and participated independently without any prior training or exposure to the task to avoid any training effect.

**Fig. 5.**
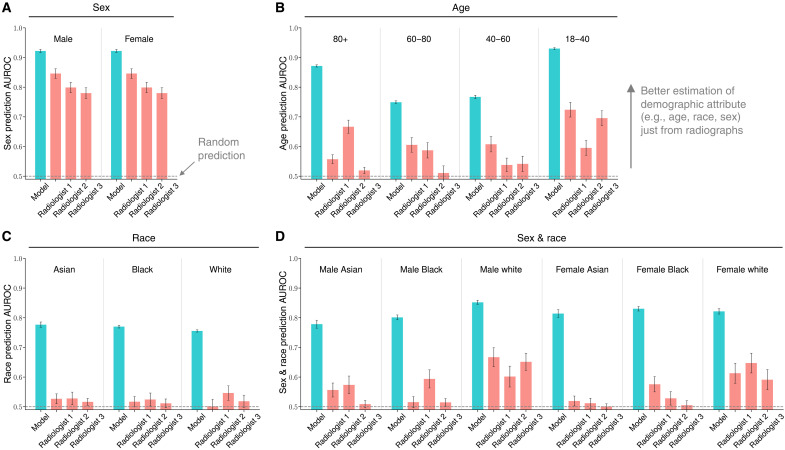
Comparisons of prediction AUROC for sensitive demographic attributes between the foundation model and three board-certified radiologists. (**A** to **D**) Prediction AUROC of subgroups within different sensitive attributes including sex (A), age (B), race (C), and the intersectional groups of sex and race (D), on a subset of MIMIC (*n* = 480). We selected out a balanced subset of MIMIC w.r.t. all attributes (i.e., balanced across age, sex, and race), and asked three board-certified radiologists to infer the attributes from just the chest x-rays. To assess the model prediction of sensitive attributes, we train a linear attribute prediction head using logistic regression on top of the penultimate layer of the model, with the model weights frozen. Error bars indicate 95% CIs estimated using nonparametric bootstrap sampling (*n* = 1000). Complete results of model predictions for other datasets are in fig. S5.

The foundation model, although trained in a self-supervised manner without explicit information regarding the demographic attributes, demonstrated substantial and consistent encoding of demographic information across all tested attributes and subgroups (Fig. 5). Specifically, the predictive AUCs for sex [female AUC = 0.92 and 95% CI (0.91 to 0.93) Fig. 5A], age [18 to 40 AUC = 0.94 and 95% CI (0.93 to 0.94); Fig. 5B], race [Black AUC = 0.78 and 95% CI (0.77 to 0.78); Fig. 5C], and the intersectional subgroups [Black female AUC = 0.83 and 95% CI (0.82 to 0.83); Fig. 5D] are significantly higher than random chance (i.e., 0.5). This strong algorithmic encoding of demographic attributes could be explainable for the observed underdiagnosis bias across patient subpopulations (details in Discussion).

However, the performance of three radiologists to predict these attributes falls behind. They achieve relatively high AUC scores in sex prediction (Fig. 5A) but much lower in age prediction (Fig. 5B). When it comes to race, the prediction is marginally better than random guess (Fig. 5C). Similar performance pattern is observed in the intersectional group of sex and race prediction (Fig. 5D), suggesting that radiologists cannot directly read attributes like age or race from radiographs. Figure S5 further suggests that inherent encoding of the sensitive data (e.g., demographics and support devices) might drive the underdiagnosis biases (details in Discussion and Materials and Methods). We provide analysis and initial methods to intervene the model fairness in figs. S6 and S7.

## DISCUSSION

We have dissected the performance of the state-of-the-art foundation model and shown consistent underdiagnosis in five internationally sourced public datasets in the chest x-ray domain. We were able to compare the results with board-certified radiologists to ground the findings. The results reveal consistently larger fairness disparities of the model compared to radiologists (Fig. 2), and that the model exhibits systematic underdiagnosis biases in marginalized subpopulations, such as female, younger, Black patients, as well as intersectional subgroups like Black female patients (Fig. 3). The demographic biases of the foundation model also persist across a wide range of unseen pathologies (Fig. 4). Further analyses show that the model encodes substantial demographic information (e.g., race), and that is significantly higher than human radiologists (Fig. 5).

Our results have multiple implications. First, the fairness-accuracy trade-off in AI models can raise complex ethical considerations (*29*, *30*). The latest advancements in medical vision-language foundation models hold the promise of a single model diagnosing countless pathologies with expert-level accuracy. Yet, our analysis shows that they exhibit substantial fairness gaps over marginalized groups. This disparity is significantly larger than that by radiologists across various diagnostic tasks and patient subpopulations (Fig. 2). Incorrectly underdiagnosing specific subgroups (e.g., Black female patients) more frequently than others not only places these individuals at a disadvantage but also raises serious ethical concerns when deploying the model in a clinical pipeline (*31*, *32*). The results have implications on the regulation of these new medical technologies (*33*), especially under the recent White House Executive Order on the safe, secure, and trustworthy development and use of AI (*34*).

Second, our study shows that the model encodes demographic information far more profoundly than human capacity (Fig. 5 and fig. S5). This suggests that inherent encoding of the sensitive data (e.g., demographics and support devices) might drive the underdiagnosis biases (e.g., Fig. 4). Notably, even though the model is trained in a self-supervised manner without explicit attribute information, it still manages to embed this information. Recent studies explore if deep models use demographics as “shortcuts,” disadvantaging specific groups (*35*, *36*). These call for a deeper understanding of how these powerful models process and utilize sensitive information, and whether that is aligned with clinical validations by radiologists. While demographics can refine differential diagnoses and are associated with patient outcomes in certain cases, they may not be a direct causal factor in most diseases (*32*, *37*) (e.g., pneumothorax, pneumonia, fracture, etc.). Whether demographic variables should be encoded as proxies for causal factors is a decision that should align with its actual clinical use (*18*, *31*, *38*).

Third, our results reveal that the AI model effectively encodes demographic information from radiographs more accurately than radiologists. A recent study demonstrates that AI can measure and interpret biological age, predict age-related outcomes, and convert these predictions into an estimated biological age (*39*). This capability extends beyond the typical scope of radiologist assessments, which generally do not include evaluating age, sex, or gender, as these details are usually obtained from electronic medical records. However, the ability of the AI model to discern these demographics more precisely suggests a potential for uncovering potentially clinically relevant features that might not be immediately apparent to human readers, suggesting an opportunity for an improved human-AI collaboration (*40*–*42*). Exploring the semantic and agnostic features harnessed by AI could improve human performance and potentially deliver a higher quality care. On the other hand, in scenarios where clinical decisions are influenced by AI model suggestions, any undetected bias within the model could lead to unintended and potentially harmful consequences (*29*, *43*). This underscores the need for careful and continuous evaluation of AI biases to progressively diminish their influence in healthcare, and ensure more accurate and equitable diagnoses.

Our study also has some limitations. First, demographics associated with the datasets are mainly self-reported or physician recorded, where inconsistencies during examinations can introduce label noise. Demographic labels can be influenced by numerous characteristics such as age, socioeconomic status, and levels of cultural assimilation (*44*–*46*). Mitigating such label noise remains challenging as traditional bias mitigation strategies may not provide effective corrections, leading to inherent biases embedded in data and models. Second, the datasets used in this study included a range of chest x-rays and projections, many of which did not adhere to a uniform standard of image quality. The extent to which this variability might have affected our results remains unclear and highlights the need for future research to explore the influence of image quality on AI model performance. Third, we focused primarily on one specific vision-language foundation model implementation. While the model is considered state-of-the-art and serves as a robust starting point, the methodologies used in the study are generic and adaptable to other foundation models (*7*–*10*). Last, while we involve human studies to assess the diagnostic performance and fairness of radiologists, they face constraints due to the limited number of participating radiologists. Expanding the scale of participants in future studies could improve the validity of the results.

In summary, we have uncovered pronounced and systematic demographic biases of the state-of-the-art visual-language foundation model, and performed human evaluations to compare them to radiologists. The results, validated across five large-scale, globally sourced datasets, showed that even when AI achieves human-level performance, deploying these algorithms in real-world scenarios demands careful consideration of the ethical implications, especially concerning underrepresented subpopulations. Furthermore, our findings prompt questions about how to understand, audit, and reduce biases in medical AI models. Developing more effective foundation models is of the utmost importance to improve diagnostic accuracy while mitigating bias and promoting equity in health care.

## MATERIALS AND METHODS

### Datasets and preprocessing

We provide additional information about the datasets used in this study. The datasets are summarized in Table 1. All five public datasets offer demographic attributes of sex and age for the associated patients. MIMIC and CheXpert additionally provide demographic information on race. To ensure the integrity of the datasets, we exclude the samples with incomplete demographic data from the dataset. Specifically, if any of the essential attributes: sex, age, or race (for MIMIC and CheXpert) is missing, the corresponding image is excluded from consideration in our study. Notably, the reported numbers in the paper regarding the datasets are post application of this exclusion criteria.

In total, we have 357,167 images from MIMIC (MIMIC-CXR), 223,458 images from CheXpert, 112,120 images from NIH (ChestX-ray14), 160,736 images from PadChest, and 5323 images from VinDr (VinDr-CXR).

#### MIMIC

The MIMIC (MIMIC-CXR) dataset (*19*) contains 357,167 chest x-rays along with free-text radiology reports, obtained from Beth Israel Deaconess Medical Center (BIDMC) in Boston, MA. The chest radiographs are retained in Digital Imaging and Communications in Medicine (DICOM) format, and the radiology reports are extracted from BIDMC electronic health record, which include textual descriptions and interpretations of the findings in the x-ray images written by radiologists during routine care.

#### CheXpert

The CheXpert dataset (*20*) consists of 223,458 chest x-rays from 64,925 patients. It offers a diverse and comprehensive collection of chest radiographs that span a wide array of subpopulations. An automated rule-based labeler was developed to extract the 14 observations (radiographic findings) from the radiology reports. Annotations from board-certified radiologists are available for both the validation set (200 studies sampled randomly from the full dataset) and test set (500 studies randomly sampled from the 1000 studies in the report evaluation set for the labeler). Three of the eight board-certified radiologists were chosen to benchmark the performance of radiologists (*20*).

#### NIH

The NIH dataset (ChestX-ray14) (*21*) is a medical imaging dataset that contains 112,120 frontal-view chest x-ray images. These images are sourced from 30,805 patients, with data collected over a large period ranging from 1992 and 2015. It provides 15 pathology labels extracted from the radiological reports through text mining techniques. As an expansion of ChestX-ray8, this dataset introduces six additional thoracic diseases: edema, emphysema, fibrosis, pleural thickening, and hernia. The chest x-ray images are resized to a resolution of 1024 × 1024 pixels from the original DICOM format.

#### PadChest

The PadChest dataset (*22*) comprises a substantial collection of 160,736 chest x-ray images obtained from 67,590 patients at San Juan Hospital (Spain) from 2009 to 2017. It offers six different radiographic projections, 174 findings, and 19 differential diagnoses. A total of 39,053 chest x-ray images were manually annotated by trained physicians, and a recurrent neural network with attention mechanism trained on the manually labeled subset was used to label the remaining samples.

#### VinDr

The VinDr (VinDr-CXR) dataset (*23*) is a public dataset comprising 5323 frontal chest x-ray images collected from 5323 patients across two major hospitals in Vietnam: Hospital 108 and Hanoi Medical University Hospital. The dataset provides labels for 27 findings (the label “other diseases” is removed from the original dataset to avoid ambiguity). Notably, it is considered a high-quality dataset of annotated images in the research community as it provides radiologists-generated annotations for all images (in both training and test sets).

### Model training and evaluation

We mainly use the CheXzero model (*10*) as a driving example to study fairness of foundation models. The vision-language model was initialized from a Vision Transformer backbone ViT-B/32 (*47*) and pretrained weights from OpenAI’s CLIP model, which excels in tasks related to vision and language understanding (*48*). The model was trained in a self-supervised manner on the MIMIC dataset with no pathology labels or annotations used, just by leveraging the radiographs with accompanying clinical texts (*10*). In addition, we also tested another vision-language foundation model, Knowledge-enhanced Auto Diagnosis (KAD) (*11*), which introduces knowledge graphs into visual-language pretraining.

We evaluated the model on our internationally sourced chest x-ray datasets. In particular, approximately 45,000 chest x-ray images used in our evaluation come with gold-standard annotations from radiologists across three datasets: CheXpert test set (666 chest x-rays with eight board-certified radiologist annotations for the presence of 14 different conditions), VinDr (5323 images with annotations from a total of 17 experienced radiologists for 27 findings and diagnoses), and a subset of PadChest (39,053 images from the original dataset annotated by trained physicians). We also tested the model performance and fairness on MIMIC (357,167 images) and NIH (112,120 images) where the labels are generated from natural language processing techniques. Following the standard preprocessing practice (*4*, *49*), we resized the radiographs to 224 × 224 and normalized them using a sample mean and SD of the dataset for model evaluation.

### Reader study details

Three board-certified radiologists from the Department of Radiology at the University of Washington, School of Medicine were tasked with evaluating demographic attributes from chest x-rays only. Each radiologist had over 10 years of experience in chest imaging, participated independently, was blinded to the demographic attributes, and received no prior training or exposure to the task to mitigate any training effect.

We used an online labeling tool (*50*) for the radiologists to create attribute labels based on the 480 preselected chest x-ray images from MIMIC. All three attribute labels are required for each image, meaning that radiologists are required to choose one label for each of the attributes: sex (female and male), age (0 to 18, 18 to 40, 40 to 60, 60 to 80, and >80), and race (Asian, Black, white, and others). Each radiologist completed this study independently and was provided with no additional information beyond the chest x-ray images themselves. The distribution of the three attributes was not disclosed to the radiologists until after they had completed the task, ensuring an unbiased evaluation process.

### Evaluation methods

To evaluate the performance of the foundation model on pathology classification, we use the following metrics: true-positive rates (TPR), true-negative rates (TNR), ROC curves, and AUC. To evaluate the underdiagnosis disparity given one demographic attribute, we use the difference in TNR (or TPR) between two specific subpopulations (e.g., Black and white patients). To evaluate and assess the learned features in the penultimate layer of the model, we use principal components analysis (*51*) to project the embeddings into a two-dimensional space for visualization.

TPR and TNR are calculated as (TP, true positive; FN, false negative; TN, true negative; and FP: false positive)

$$\text{TPR}=\frac{\text{TP}}{\text{TP}+\text{FN}}$$

$$\text{TNR}=\frac{\text{TN}}{\text{TN}+\text{FP}}$$

We plotted ROC curves that demonstrate the trade-off between TPR and TNR as the classification thresholds are varied. When reporting the TPR and TNR, we used the optimal threshold computed on the validation set that maximizes the Youden’s *J* statistic (*25*). We followed standard nonparametric bootstrap sampling (*n* = 1000) to calculate the 95% CI (*52*). We also reported AUC, which is the area under the corresponding ROC curves showing an aggregate measure of detection performance.

### Assessing the demographic fairness of the model

To measure the fairness of the foundation model, we evaluate the metrics described above for each demographic subpopulation (defined over demographic attributes including sex, age, and race), and the differences in metric outcomes across these groups. The principle of equal TPR and TNR across different demographic subgroups is known as equal odds (*24*), a concept well-established in algorithmic fairness (*53*, *54*). Given that the models we investigate in this work will likely be used as screening or triage tools, it is crucial to recognize that the cost of an FP may vary considerably from that of an FN. Specifically, for a specific pathology, FNs [corresponding to underdiagnosis (*14*)] would be more costly than FPs, and so we focus on the FNR (or TPR) for this task. For the task of no finding prediction, we focus on the FPR (or TNR) for the same reason. Equality in one of the class conditioned error rates is an instance of equal opportunity (*24*). Consequently, we calculate underdiagnosis disparity as the difference in TNR (or TPR) between two selected subgroups.

In addition, we provide results for overdiagnosis (Fig. 3). For the no finding label, FNR is used for overdiagnosis. Similar observations hold for overdiagnosis (Fig. 3, C and D), the gaps become more substantial between intersectional subgroups.

### Prompt design

In the main paper, we primarily use prompts for vision-language foundation models for zero-shot inference, which involves calculating the similarity between x-ray representations and text representations for zero-shot classification. In particular, we follow established literature (*7*, *8*, *10*) to design the standard prompts for the vision-language foundation model.

**Zero-shot classification, radiological findings (Fig. 1, and other main figures)**

The patient has {pathology / no pathology}

Example: “The patient has pneumonia” & “The patient has no pneumonia”

**Zero-shot classification, radiological findings, with attribute info (fig. S7)**

The {attribute} patient has {pathology / no pathology}

Example: “The female patient has pneumonia”

Example: “The Black patient has no lung opacity”

Example: “The age over 80 patient has cardiomegaly”

**Zero-shot classification, demographic attributes (fig. S6)**

The patient’s gender is {attribute}

Example: “The patient patient’s gender is female”

Example: “The patient patient’s gender is male”

The patient’s age is {attribute}

Example: “The patient patient’s age is under 18”

Example: “The patient patient’s age is between 18 and 40”

Example: “The patient patient’s age is between 40 and 60”

Example: “The patient patient’s age is between 60 and 80”

Example: “The patient patient’s age is over 80”

The patient’s race is {attribute}

Example: “The patient patient’s race is Asian”

Example: “The patient patient’s race is Black”

Example: “The patient patient’s race is white”

Example: “The patient patient’s race is neither white, Black, nor Asian”

### Additional evaluation results

#### Assessing the encoding of attributes by text prompts

We assessed the algorithmic encoding of demographic attributes in the foundation model through a logistic regression layer on the top of the model embedding (Fig. 5). Since the foundation model also supports textual prompts as input, we assess the encoding again by directly using textual prompts (fig. S6). Specifically, we used prompts containing demographic information (e.g., “The patient’s gender is male.”) to assess the attribute prediction accuracy. Across different datasets, the resulting prediction AUC is lower than using logistic regression, but still significantly higher than random chance over most of the subgroups.

#### Model fairness intervention

We conducted experiments to explore fairness intervention of the foundation model by incorporating demographic details into the input prompt (fig. S7). We proposed to intervene the model prediction over subgroups by including demographic information in the input texts (e.g., “Does this female patient have pneumonia?”). Figure S7 shows complex outcomes: After such intervention, the model displays reduced demographic biases for certain conditions like lung opacity and no finding (fig. S7, A and C), but not for others like “pneumonia” (fig. S7B). The results indicate that it is possible to improve the demographic fairness of the model while maintaining the overall performance, but deeper analyses are needed for more principled methods.

#### Quantifying the distribution differences between subgroups

We follow (*36*, *54*) to perform a series of hypothesis tests on the MIMIC-CXR dataset to determine whether there are statistical differences in distributions between demographic groups. These tests were inspired by prior research (*54*), and all *P* values were adjusted for multiple testing using Bonferroni correction (*54*). Specifically, we consider prevalence shifts as defined by the total variational distance between the probability distributions of Y conditioned on different groups, and representation shifts as defined by the mean maximum discrepancy distance in their distribution of representations across demographic groups. As tables S1 and S2 indicate, both significant prevalence and representation shifts are observed between subgroups.

#### Comparisons between self-supervised and supervised learning models

We conducted a comparative analysis of the self-supervised foundation model with a fully supervised model regarding demographic fairness. Specifically, we selected a state-of-the-art supervised baseline model with a DenseNet backbone, as described in the literature (*12*, *14*, *54*). This supervised model achieves comparable overall area under the receiver operating characteristic curve (AUROC) to the self-supervised foundation model (AUROC of no finding on MIMIC test set: supervised, 0.84; and self-supervised, 0.85). Table S3 summarizes the fairness disparities, where on the MIMIC test set (unseen during training), the self-supervised model exhibited lower fairness gaps consistently across attributes and tasks. When tested on external datasets (CheXpert, NIH, PadChest, and VinDr), the supervised model generally achieved lower gaps compared to the self-supervised model. These results highlight the complex nature of fairness in real-world setups and under distribution shifts (*54*).

### Statistical analysis

#### Underdiagnosis disparity

One-tailed Wilcoxon rank-sum test (α = 0.05) was used to assess the underdiagnosis disparity between the foundation model and radiologists.

#### AUROC

We collect AUROC results for pathology prediction across five datasets using the foundation model’s predictions. We also present the AUROC results for pathology prediction from external board-certified radiologists on the CheXpert test set (*n* = 666) and VinDr test set (*n* = 5323). We collect AUROC results for attribute prediction (e.g., race) across five datasets using the foundation model’s predictions with textual input changed to attribute predictions. We also present the AUROC results for attribute prediction from three board-certified radiologists on the subset of MIMIC (*n* = 480). The 95% CI for the true AUCs were estimated using nonparametric bootstrap sampling (*n* = 1000).

#### TPR and TNR

The mean metric is reported along with 95% Cis, which are estimated using nonparametric bootstrap sampling (*n* = 1000).

#### Confidence intervals

We use the nonparametric bootstrap sampling to generate CIs: random samples of size *n* (equal to the size of the original dataset) are repeatedly sampled 1000 times from the original dataset with replacement. We then estimate the AUC, subgroup TNR (or TPR), and underdiagnosis disparity (fairness gaps) metrics using each bootstrap sample (α = 0.05). All statistical analysis was performed with Python version 3.9 (Python Software Foundation).
