# Supplementary material for: Demographic bias of expert-level vision-language foundation models in medical imaging
Source: Sci Adv. 2025 Mar 26;11(13):eadq0305. doi: 10.1126/sciadv.adq0305 (PMC11939055; doi:10.1126/sciadv.adq0305)
Supplement: Supplementary file 1 — Figs. S1 to S7 Tables S1 to S3 [file sciadv.adq0305_sm.pdf]

Science Advances
AAAS

Supplementary Materials for

Demographic bias of expert-level vision-language foundation models in medical imaging

Yuzhe Yang *et al.*

Corresponding author: Yuzhe Yang, yuzheyangpku@gmail.com

Sci. Adv. **11**, eadq0305 (2025)
DOI: 10.1126/sciadv.adq0305

This PDF file includes:

Figs. S1 to S7
Tables S1 to S3

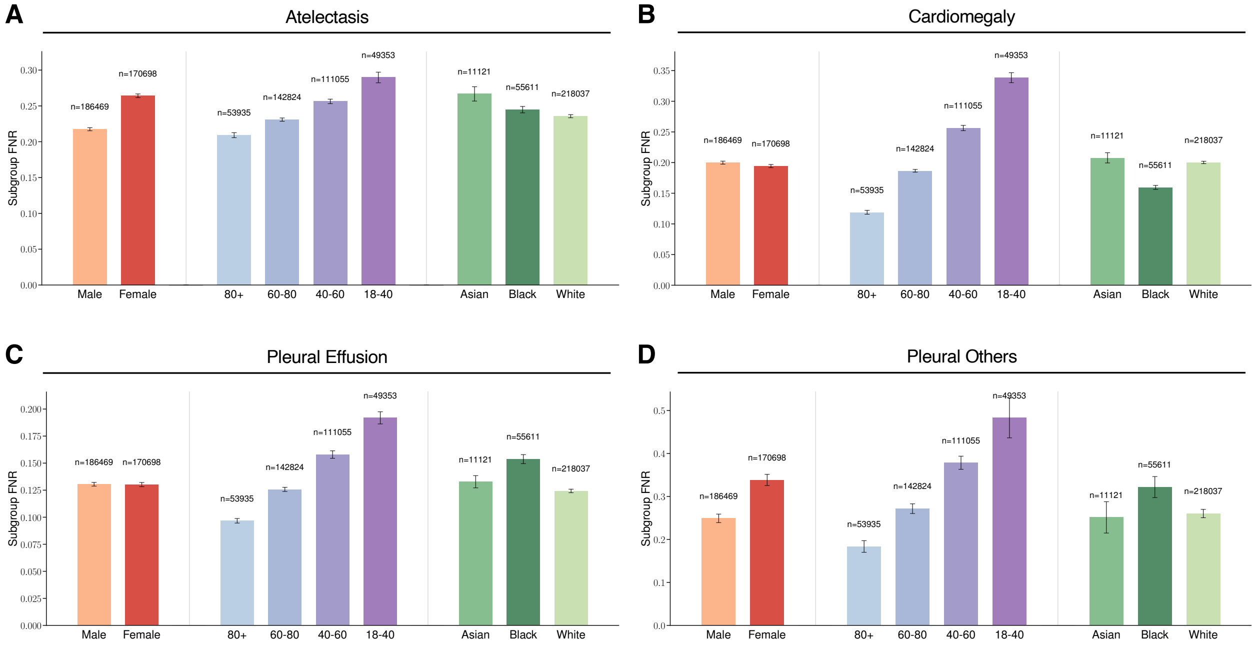

Fig. S1. Underdiagnosis disparities on different pathologies of another vision-language foundation model, KAD (*11*), across subgroups of sex, age, and race in the MIMIC dataset. (**A** to **D**) The underdiagnosis rate for "Atelectasis", "Cardiomegaly", "Pleural Effusion", and "Pleural Others" in the indicated patient subpopulations. Error bars indicate 95% confidence intervals estimated using non-parametric bootstrap sampling (n=1,000).

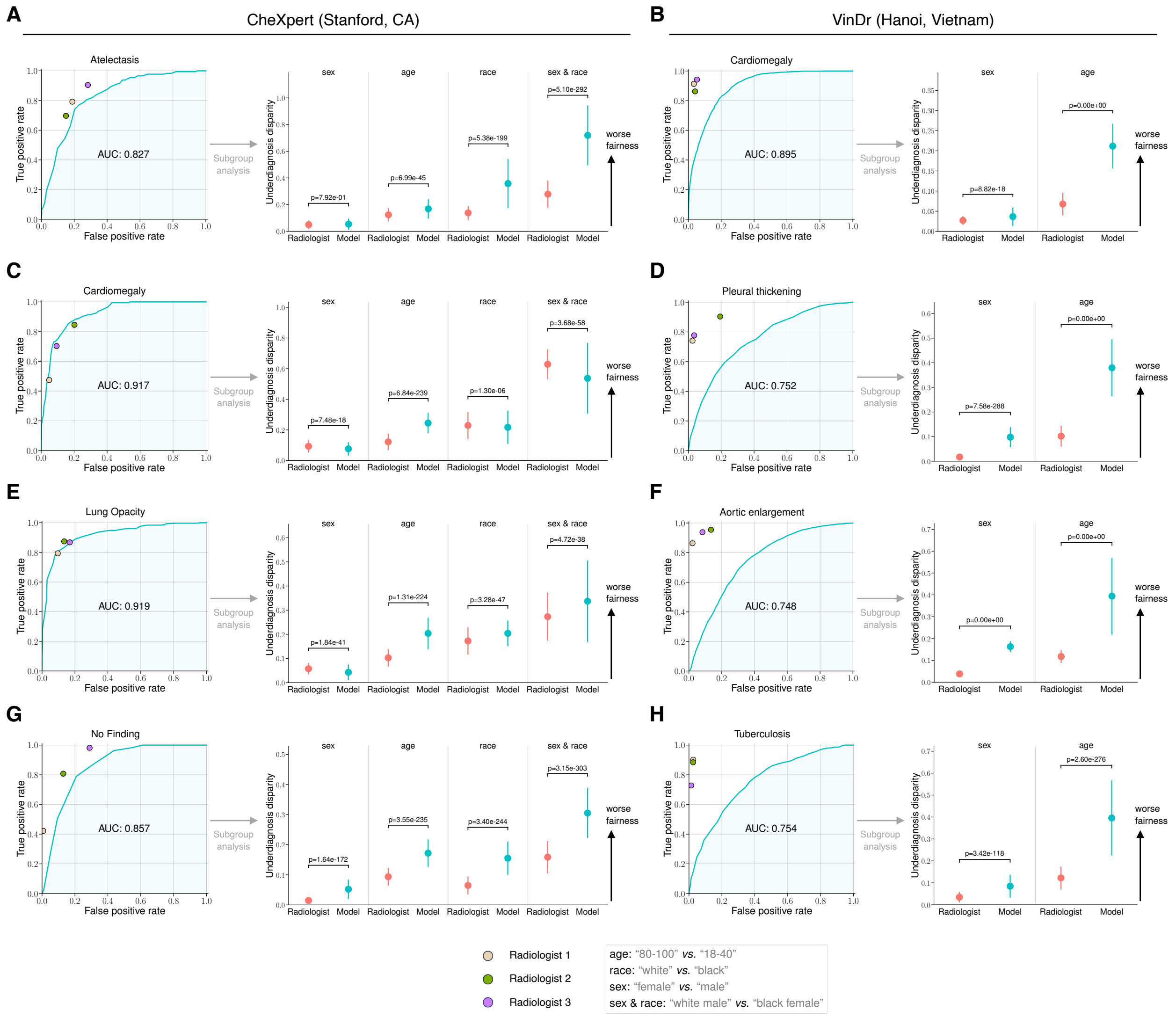

Fig. S2. Comparisons of diagnosis AUROC and underdiagnosis disparity for the vision-language foundation model and board-certified radiologists on different datasets. (**A, C, E, G**) Comparison of the ROC curve (left) and the underdiagnosis disparity (right) of the model to benchmark radiologists against the test-set ground truth on the CheXpert dataset (n=666). (**B, D, F, H**) The same comparisons performed on another dataset from a different country, VinDr (n=5,323). We average the assessments from different radiologists as the evaluation of human biases. The model exhibits significantly higher underdiagnosis bias than that of radiologists on all three pathologies. Error bars indicate 95% confidence intervals estimated using non-parametric bootstrap sampling (n=1,000).

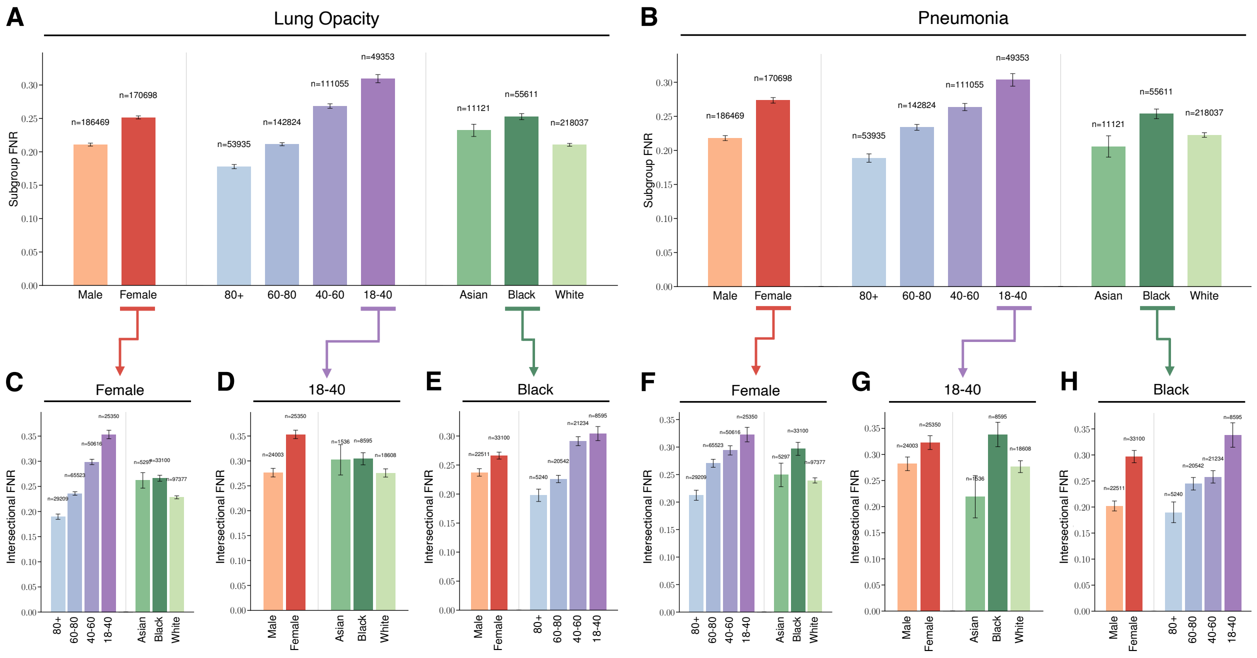

Fig. S3. Underdiagnosis disparities on different pathologies across subgroups of sex, age, race, and intersectional groups in the MIMIC dataset. (**A**) The underdiagnosis rate for "Lung Opacity" in the indicated patient subpopulations. (**B**) The underdiagnosis rate for "Pneumonia" in the indicated patient subpopulations. (**C** to **E**) Intersectional underdiagnosis rates for "Lung Opacity" in female patients (C), patients aged 18–40 years (D), and Black patients (E). (**F** to **H**) Intersectional underdiagnosis rates for "Pneumonia" in female patients (F), patients aged 18–40 years (G), and Black patients (H). Error bars indicate 95% confidence intervals estimated using non-parametric bootstrap sampling (n=1,000).

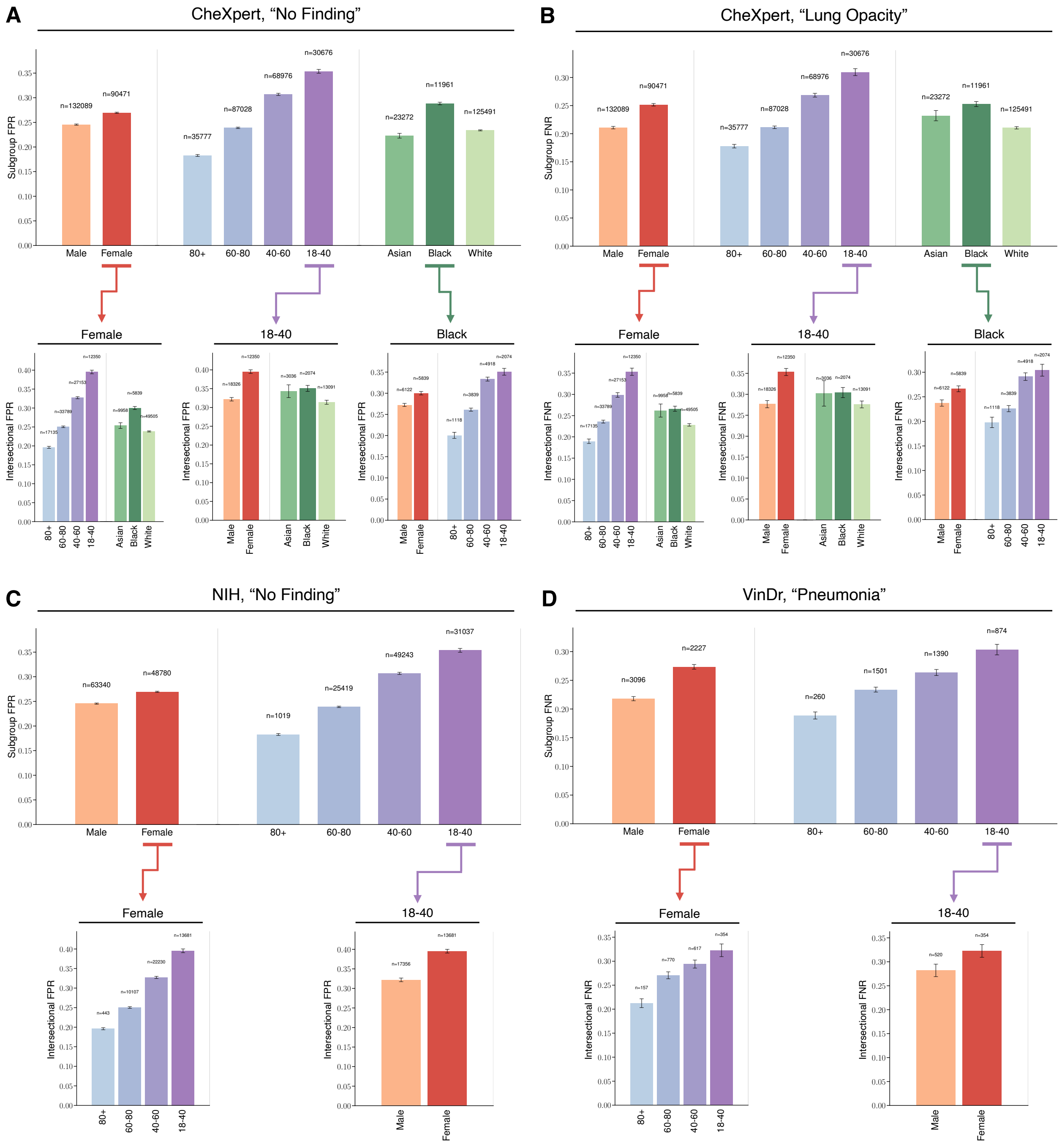

Fig. S4. Underdiagnosis disparities on different pathologies across subgroups of sex, age, race, and intersectional groups in CheXpert, NIH, and VinDr. (**A**) The underdiagnosis rate for “No Finding” in CheXpert in the indicated patient subpopulations. (**B**) The underdiagnosis rate for “Lung Opacity” in CheXpert in the indicated patient subpopulations. (**C**) The underdiagnosis rate for “No Finding” in NIH in the indicated patient subpopulations. (**D**) The underdiagnosis rate for “Pneumonia” in VinDr in the indicated patient subpopulations. Error bars indicate 95% confidence intervals estimated using non-parametric bootstrap sampling (n=1,000).

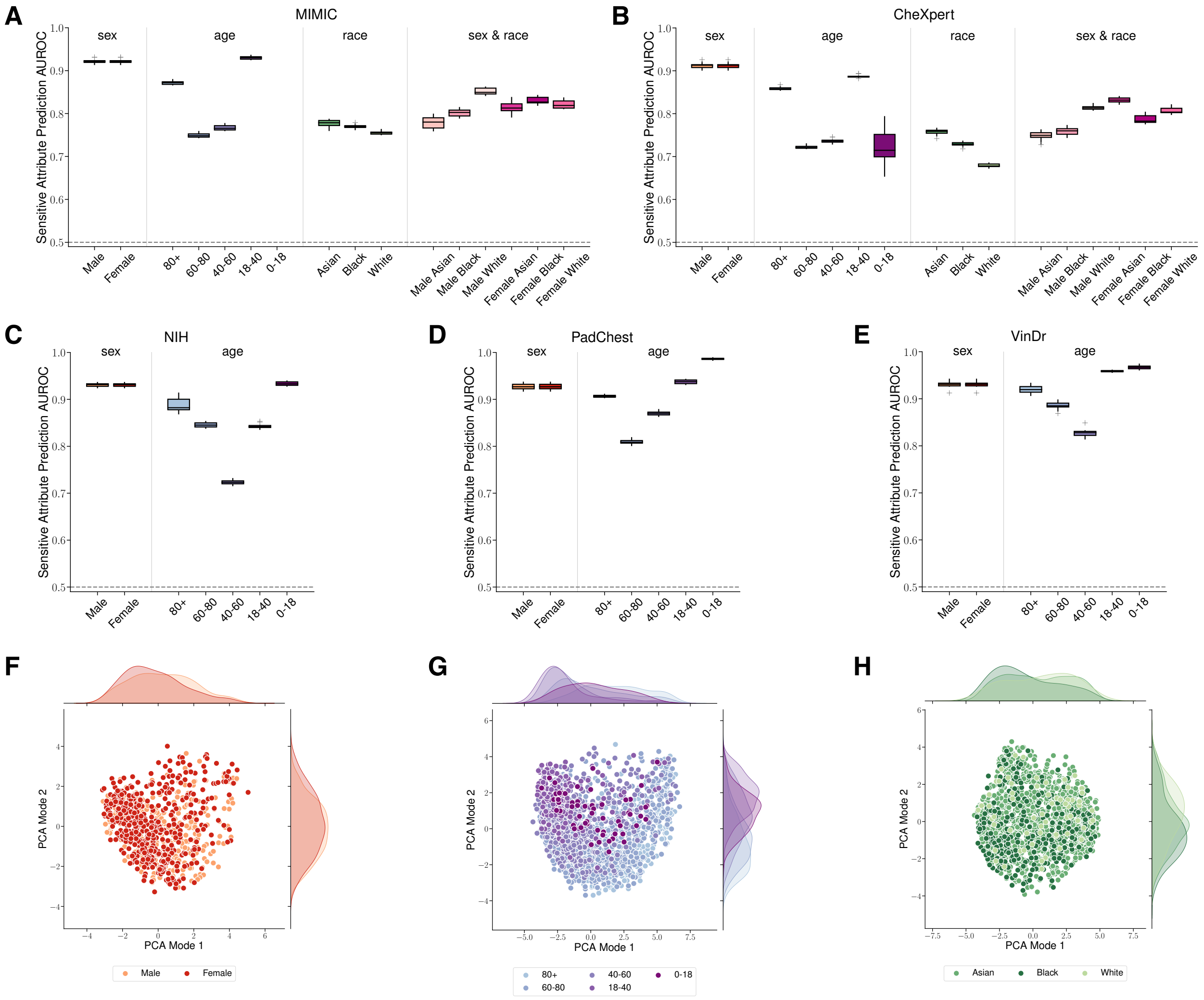

Fig. S5. Algorithmic encoding of sensitive attributes in the foundation model. (**A** to **E**) Prediction AUROC of different sensitive attributes including age, sex, race, and intersectional groups, across five datasets including MIMIC (A), CheXpert (B), NIH (C), PadChest (D), and VinDr (E). We train a linear attribute prediction head using logistic regression on top of the penultimate layer of the model, with the model weights frozen. (**F** to **H**) PCA visualization of the learned features in the penultimate layer of the model. We visualize the feature distribution on the randomly subsampled CheXpert dataset (n=2,000) for different attributes including sex (F), age (G), and race (H).

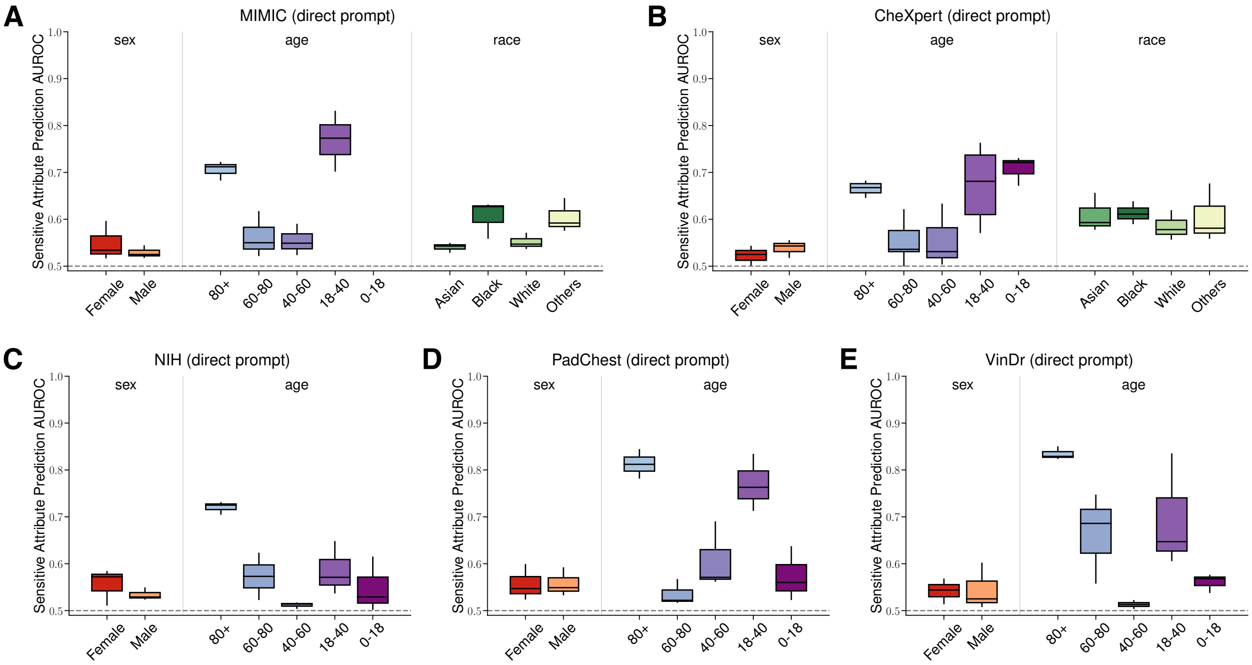

Fig. S6. Direct Attribute prediction AUROC of the foundation model across different datasets. (**A** to **E**) We utilize textual prompts encompassing demographic information (e.g., "The patient's gender is male."; details in Methods) to assess the attribute prediction accuracy on the MIMIC (A), CheXpert (B), NIH (C), PadChest (D), and VinDr (E) datasets. Error bars indicate 95% confidence intervals estimated using non-parametric bootstrap sampling (n=1,000).

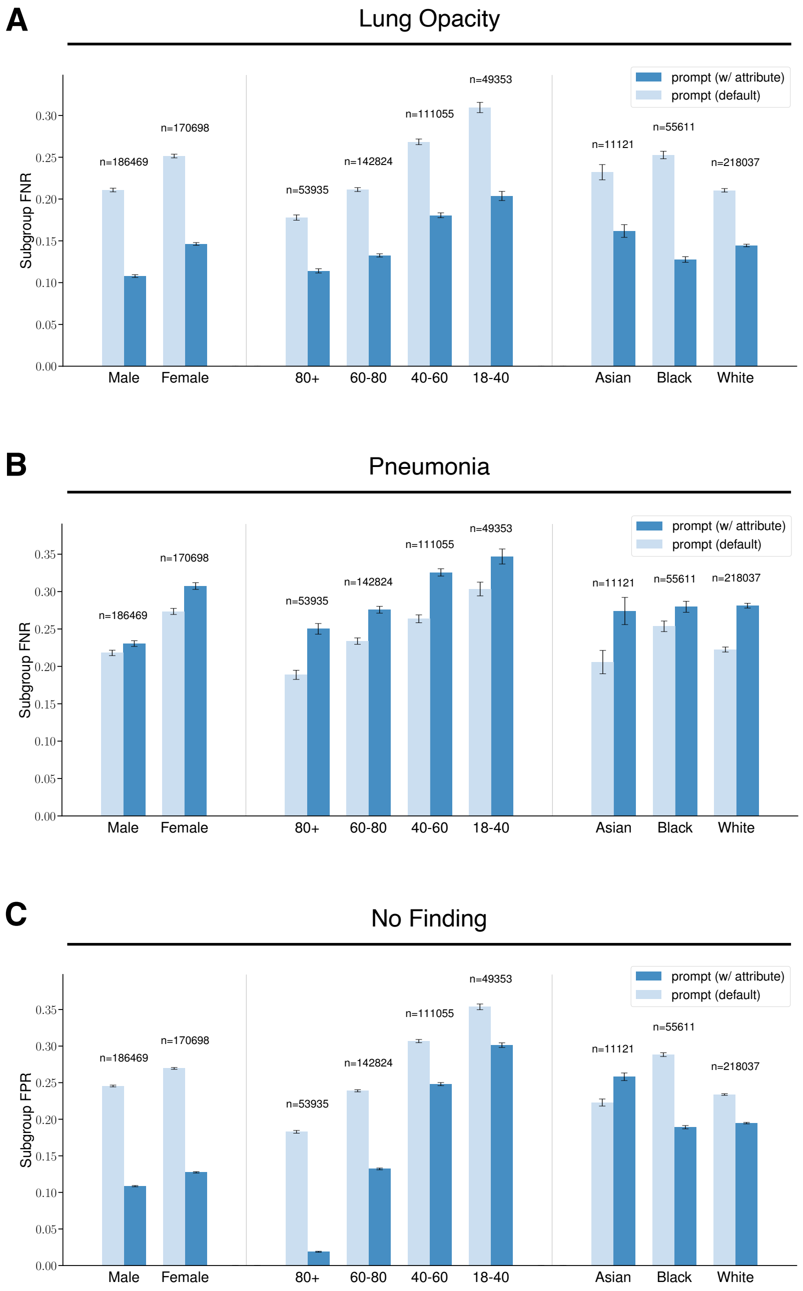

Fig. S7. Model fairness intervention by incorporating demographic details into the input prompt. (**A** to **C**) Performance across subgroups before and after introducing the sensitive demographic details into the prompt, for "Lung Opacity" (A), "Pneumonia" (B), and "No Finding" (C). We proposed to intervene the model prediction over subgroups by including demographic information in the input texts (e.g., "Does this female patient have Pneumonia?"; details in Methods). After this intervention, the model displays reduced demographic biases for certain conditions like "Lung Opacity", but not for others like "Pneumonia". Error bars indicate 95% confidence intervals estimated using non-parametric bootstrap sampling (n=1,000).

Table S1. Distribution differences quantification between demographic subgroups in the MIMIC dataset. (**A**) Prevalence shift P(Y|A) was derived using the total variational distance between the probability distributions of Y conditioned on different groups. P values were computed using a two-sided proportion z-test. (**B**) Representation shift P(X|A) was derived by encoding input into representations from a frozen foundation model (i.e., CheXzero (*10*)) and computing the MMD distance with a Gaussian kernel (*54*). P values were computed using a two-sided permutation test using this distance as the test statistic (*54*).

A Prevalence shift: $P(Y|A=a_1)$ vs. $P(Y|A=a_2)$

**Attribute**	**Group 1**	**Group 2**	**Distance**	p **value**†
Sex	Female	Male	0.054	***
Age	40-60	18-40	0.187	***
	80-100	40-60	0.221	***
	80-100	60-80	0.083	***
	80-100	18-40	0.408	***
	60-80	40-60	0.138	***
	60-80	18-40	0.325	***
Race	White	Black	0.097	***
	White	Other	0.176	***
	White	Asian	0.005	not significant
	Black	Other	0.079	***
	Black	Asian	0.092	***
	Asian	Other	0.171	***

† Bonferroni correction for multiple testing. *** indicates $p < 0.001$.

B Representation shift: $P(X|A=a_1)$ vs. $P(X|A=a_2)$

**Attribute**	**Group 1**	**Group 2**	**Distance**	p **value**†
Sex	Female	Male	0.003	***
Age	40-60	18-40	0.016	***
	80-100	40-60	0.020	***
	80-100	60-80	0.004	***
	80-100	18-40	0.063	***
	60-80	40-60	0.007	***
	60-80	18-40	0.040	***
Race	White	Black	0.004	***
	White	Other	0.008	***
	White	Asian	0.002	***
	Black	Other	0.004	***
	Black	Asian	0.004	***
	Asian	Other	0.006	***

† Bonferroni correction for multiple testing. *** indicates $p < 0.001$.

Table S2. Prevalence rates of representative tasks across different datasets for each demographic subgroup used in this study.

		MIMIC				CheXpert			
		**Cardiomegaly**	**No Finding**	**Effusion**	**Pneumothorax**	**Cardiomegaly**	**No Finding**	**Effusion**	**Pneumothorax**
**Sex (%)**	**Female**	15.1	42.6	18.9	2.8	11.6	10.2	38.8	8.3
	**Male**	14.8	37.2	21.1	4.0	12.4	9.9	38.4	9.0
**Race (%)**	**Asian**	16.6	36.0	24.2	5.4	12.7	10.4	40.5	9.8
	**Black**	17.6	44.3	13.4	1.8	19.6	11.7	31.7	5.8
	**White**	15.5	34.6	24.0	4.0	11.5	9.4	39.4	9.1
	**Other**	11.1	52.5	12.6	2.5	11.7	10.8	37.6	8.0
**Age (%)**	**18-40**	6.8	64.0	8.1	3.6	9.1	20.5	27.0	12.5
	**40-60**	11.4	46.5	15.0	3.0	10.1	12.4	36.2	8.6
	**60-80**	17.6	32.5	23.9	3.8	12.4	7.0	42.3	8.9
	**80-100**	22.9	23.3	31.0	3.0	17.9	3.7	44.2	5.0
**Intersection (%)**	**Asian Female**	16.9	38.6	22.7	4.7	12.5	10.7	40.5	9.8
	**Asian Male**	16.4	33.6	25.5	6.1	12.8	10.1	40.4	9.8
	**Black Female**	18.3	46.6	13.0	1.5	20.9	11.5	31.8	4.7
	**Black Male**	16.7	41.0	13.9	2.2	18.2	11.9	31.6	6.8
	**White Female**	15.5	36.3	23.5	3.5	10.2	9.4	39.9	9.0
	**White Male**	15.4	33.3	24.4	4.4	12.4	9.4	39.0	9.2
	**Others Female**	10.7	57.2	10.9	1.7	12.1	11.4	37.6	7.0
	**Others Male**	11.5	48.1	14.2	3.3	11.4	10.4	37.6	8.7

Table S3. Comparisons in demographic fairness between self-supervised foundation models and supervised learning models. (**A** to **B**) We compare vision-language foundation models with state-of-the-art supervise learning models (*10*, *54*) across different datasets and attributes, on "No Finding" (A) and "Cardiomegaly" (B) prediction.

A No Finding

	MIMIC†				CheXpert				NIH		PadChest		VinDr	
	sex	age	race	sex & race	sex	age	race	sex & race	sex	age	sex	age	sex	age
Self-supervised	**2.4%**	**17.1%**	**5.5%**	**7.0%**	4.8%	**5.9%**	11.8%	5.9%	2.2%	**3.4%**	**4.2%**	77.9%	10.2%	15.2%
Supervised	3.8%	31.1%	8.1%	10.1%	**0.1%**	17.1%	**2.0%**	**1.8%**	**1.3%**	9.8%	4.8%	**41.6%**	**1.3%**	**8.2%**

† MIMIC is the in-distribution (ID) dataset. Both models achieve high AUROC on the ID test set (supervised: 0.85; self-supervised: 0.84).

B Cardiomegaly

	MIMIC†				CheXpert				NIH		PadChest		VinDr	
	sex	age	race	sex & race	sex	age	race	sex & race	sex	age	sex	age	sex	age
Self-supervised	**0.6%**	22.0%	**4.1%**	**4.3%**	7.0%	13.4%	**6.8%**	**5.2%**	**4.9%**	**1.9%**	**2.9%**	50.3%	3.9%	7.5%
Supervised	2.3%	**20.5%**	6.8%	7.2%	**2.6%**	**7.0%**	13.4%	8.5%	6.3%	22.4%	8.4%	**45.2%**	**3.0%**	**2.2%**

† MIMIC is the in-distribution (ID) dataset. Both models achieve high AUROC on the ID test set (supervised: 0.84; self-supervised: 0.82).
